# Masquerade Syndrome Presenting as Optic Neuritis and Retinitis Due to Pituitary Germinoma

**DOI:** 10.7759/cureus.115964

**Published:** 2026-09-08

**Authors:** Atsuki Fukushima, Yoshitake Kato, Hitoshi Tabuchi

**Affiliations:** 1 Ophthalmology, Tsukazaki Hospital, Himeji, JPN; 2 Ophthalmology, Hiroshima University, Hiroshima, JPN

**Keywords:** germinoma, hydrocephalus, masquerade syndromes, neuroretinitis, uveitis

## Abstract

Masquerade syndrome is a collective term for various ocular diseases presenting with clinical features resembling intraocular inflammation. Delays in definitive diagnosis and treatment can not only lead to irreversible vision loss but also impact the patient's prognosis regarding survival. We report the case of a 26-year-old male patient who presented with impaired vision in the right eye, which had persisted for six months; neuroretinitis was diagnosed. Although infectious neuroretinitis was suspected, tests for pathogens such as *Mycobacterium tuberculosis*, *Toxoplasma*, *Toxocara*, and *Bartonella* were all negative. Treatment with betamethasone eye drops alone resulted in a fluctuating course of improvement and worsening of the neuroretinitis. Ten months after the initial visit, the patient's headache-which had begun one month prior-worsened and was accompanied by vomiting and a pulsating sensation, leading to a consultation with our department of neurosurgery. Magnetic resonance imaging (MRI) revealed a lobulated mass extending from the right midbrain to the right thalamus, causing narrowing of the cerebral aqueduct and hydrocephalus. Four days after the neurosurgical consultation, the patient underwent endoscopic third ventriculostomy (ETV) and a brain biopsy, leading to a definitive diagnosis of germinoma. The headache improved the day after surgery, and by one month after the operation, the hydrocephalus had resolved, while neuroretinitis also showed improvement. The patient received carboplatin and etoposide (CARE) treatment in the second, third, and fourth months post-surgery, whole-ventricle radiotherapy (24 Gy/15 fractions) in the fifth and sixth months, and ifosfamide (IFO), cisplatin (CDDP), and etoposide (VP-16) (ICE) treatment in the seventh, ninth, and 11th months. No recurrence of the brain tumor has been observed since then. At the final follow-up visit five years post-surgery, the neuroretinitis had resolved. When neuroretinitis is observed in a young patient and infectious causes have been ruled out, intracranial germinoma should be considered in the differential diagnosis.

## Introduction

Masquerade syndromes are a diverse group of ocular disorders that present with clinical features resembling intraocular inflammation but arise from underlying conditions that are not primarily inflammatory diseases. Because they closely mimic uveitis-the prototypical ocular inflammatory disease-diagnosis is often challenging. Misdiagnosing the condition as uveitis and administering inappropriate immunosuppressive therapy can delay the correct diagnosis and appropriate treatment; this may result not only in irreversible vision loss but also in consequences affecting the patient's prognosis for survival [1]. While reports indicate that non-malignant conditions can also present with similar clinical features [2], malignant neoplasms are the most frequently reported causes [1,3,4]. Among these, malignant lymphoma is a representative cause of masquerade syndromes [5].

Although infrequent, cases of masquerade syndrome caused by germinoma have been reported [6-10]. Thus, masquerade syndrome caused by germinoma appears to be rare. It has become evident that the clinical presentation is characterized by neuroretinitis in patients with masquerade syndrome due to germinoma.

## Case presentation

The patient was a 26-year-old man who presented with a chief complaint of impaired vision in the right eye persisting for six months. At the initial visit, visual acuity was 0.6 (1.5) in the right eye and 1.5 (1.5) in the left eye, and intraocular pressure was approximately 15 mmHg in both eyes. The mean critical flicker frequency (CFF) values were 42 Hz in the right eye and 46 Hz in the left eye. Although anterior chamber inflammation was mild, findings of vitritis, retinal vasculitis, optic disc hyperemia and swelling, and retinal exudates were observed; a diagnosis of neuroretinitis was made (Figure 1).

**Figure 1:**
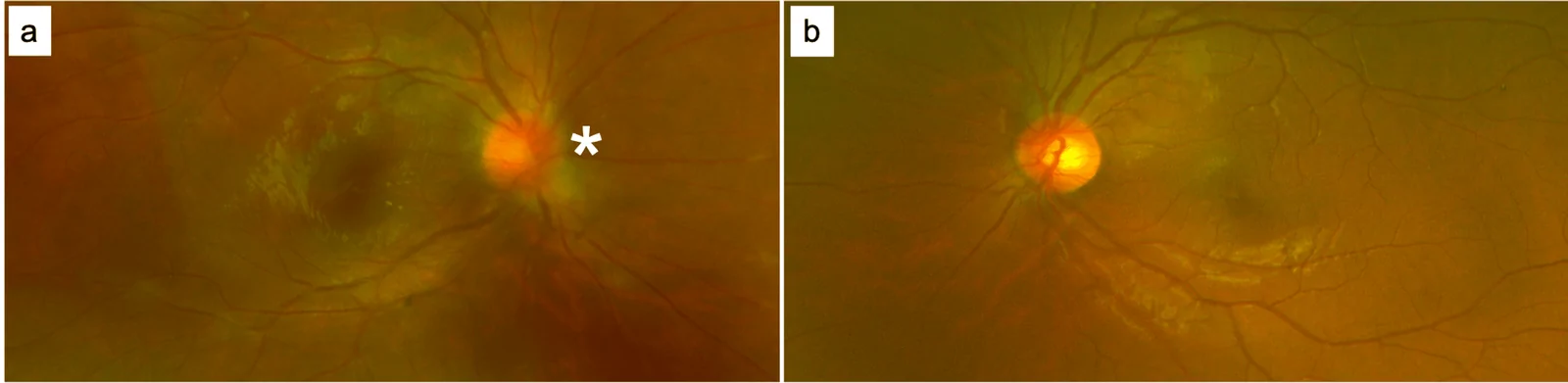
Color fundus photographs at the initial visit (a) Right eye; (b) left eye. The image of the right eye is blurred; redness, swelling, and blurred margins of the optic disc were observed (white asterisk).

Optical coherence tomography (OCT) revealed a serous retinal detachment at the right fovea (Figure 2).

**Figure 2:**
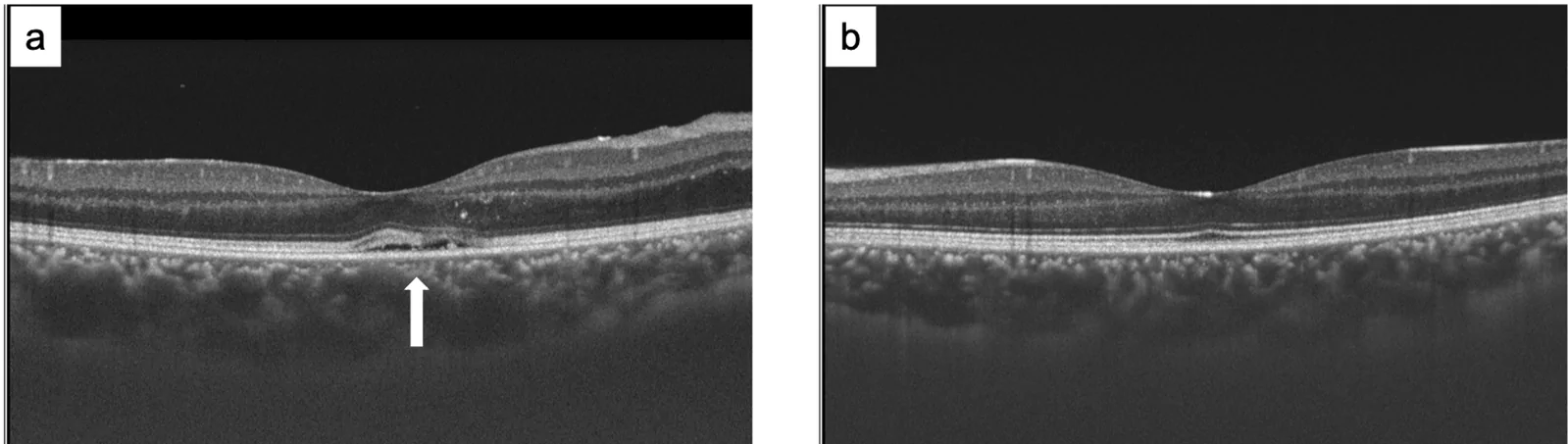
Optical coherence tomography (OCT) images at the initial visit (a) Right eye; (b) left eye. Serous retinal detachment was observed at the fovea of the right eye (white arrow).

To rule out infectious causes, tests for tuberculosis, *Toxoplasma*, *Toxocara*, and *Bartonella* were performed, but all results were negative. No systemic medication was administered; treatment consisted solely of betamethasone eye drops, and the neuroretinitis followed a course of alternating improvement and exacerbation. Fundus photography (Figure 3), fluorescein angiography (Figure 4), and OCT (Figure 5) performed six months after the initial visit revealed severe signs of inflammation in the left eye-an eye that had shown few abnormalities at the time of the initial presentation.

**Figure 3:**
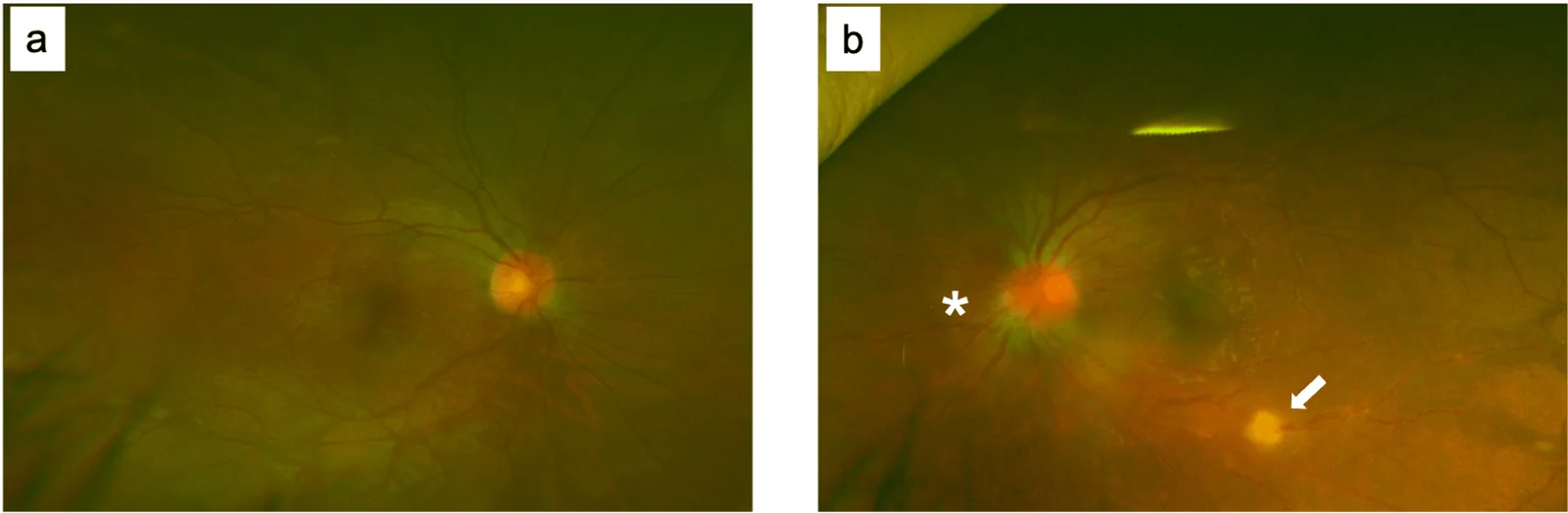
Color fundus photographs six months after the initial visit (a) Right eye; (b) left eye. Redness, swelling, and blurred margins of the optic disc were observed in the left eye (white asterisk). Additionally, a circular exudate was noted along the inferior arcade vessel (white arrow). The right eye showed improvement compared to the initial visit (Figure 1a).

**Figure 4:**
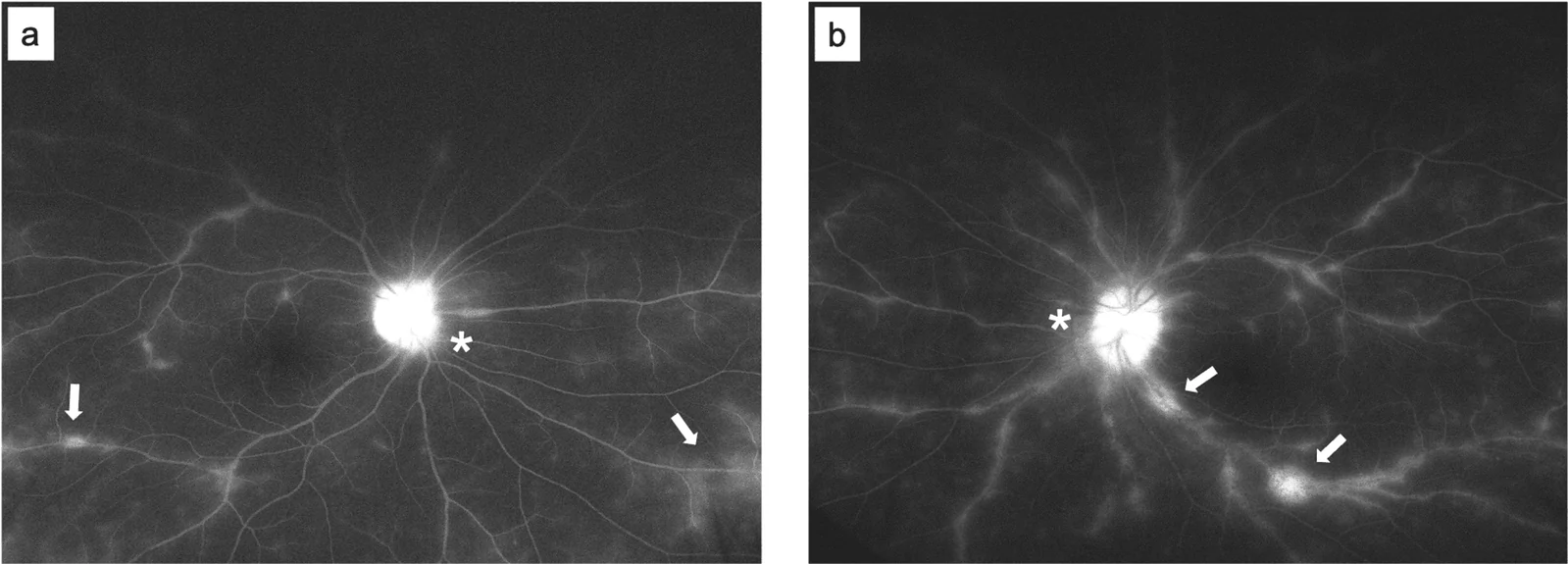
Fluorescein angiography images six months after the initial visit (a) Right eye; (b) left eye. Fluorescein staining and leakage at the optic disc were observed in both eyes (white asterisks). Fluorescein leakage from retinal vessels was also observed in both eyes (white arrows).

**Figure 5:**
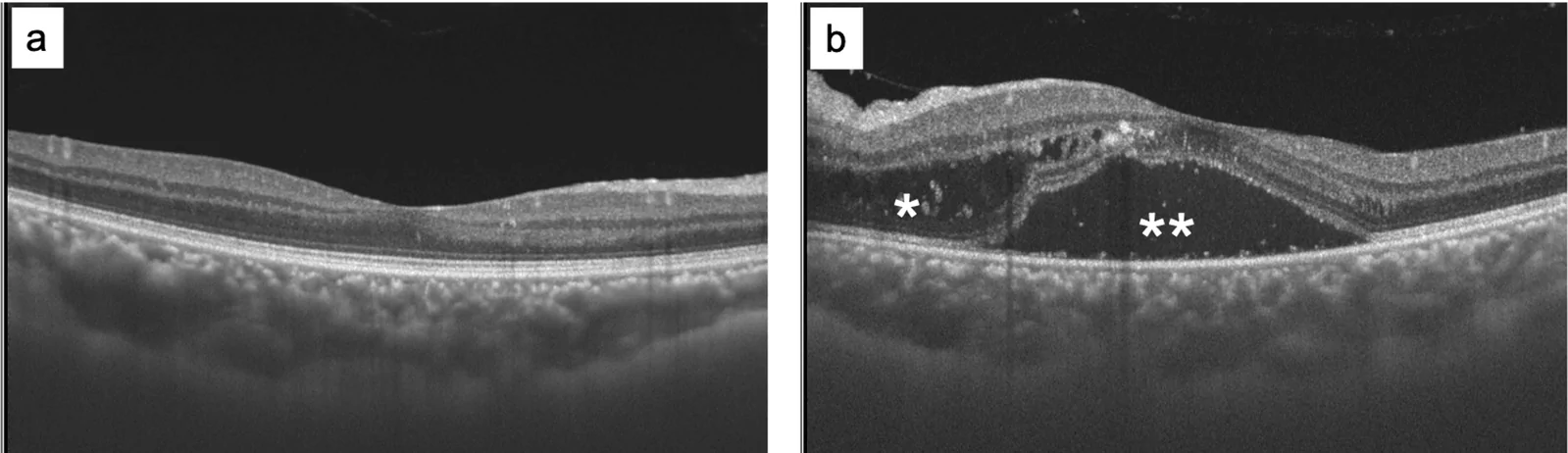
Optical coherence tomography (OCT) images six months after the initial visit (a) Right eye; (b) left eye. Serous retinal detachment (two white asterisks) and intraretinal fluid (a single white asterisk) were observed at the fovea of the left eye. The right eye showed improvement compared to the initial visit (Figure 2a).

Conversely, findings in the right eye (Figures 3, 5) showed improvement compared to the initial examination (Figures 1, 2). Ten months after the initial visit, the patient presented to our neurosurgery department due to a headache that had worsened over the past month, accompanied by vomiting and a pulsating sensation starting three days prior. Magnetic resonance imaging (MRI) revealed narrowing of the cerebral aqueduct and hydrocephalus caused by a lobulated mass extending from the right midbrain to the right thalamus (Figure 6a). Four days after the neurosurgical consultation, the patient underwent endoscopic third ventriculostomy (ETV) and a brain biopsy, resulting in a definitive diagnosis of germinoma. The headache improved the day after surgery; one month post-operatively, the hydrocephalus had resolved (Figure 6b), and the neuroretinitis also gradually improved.

**Figure 6:**
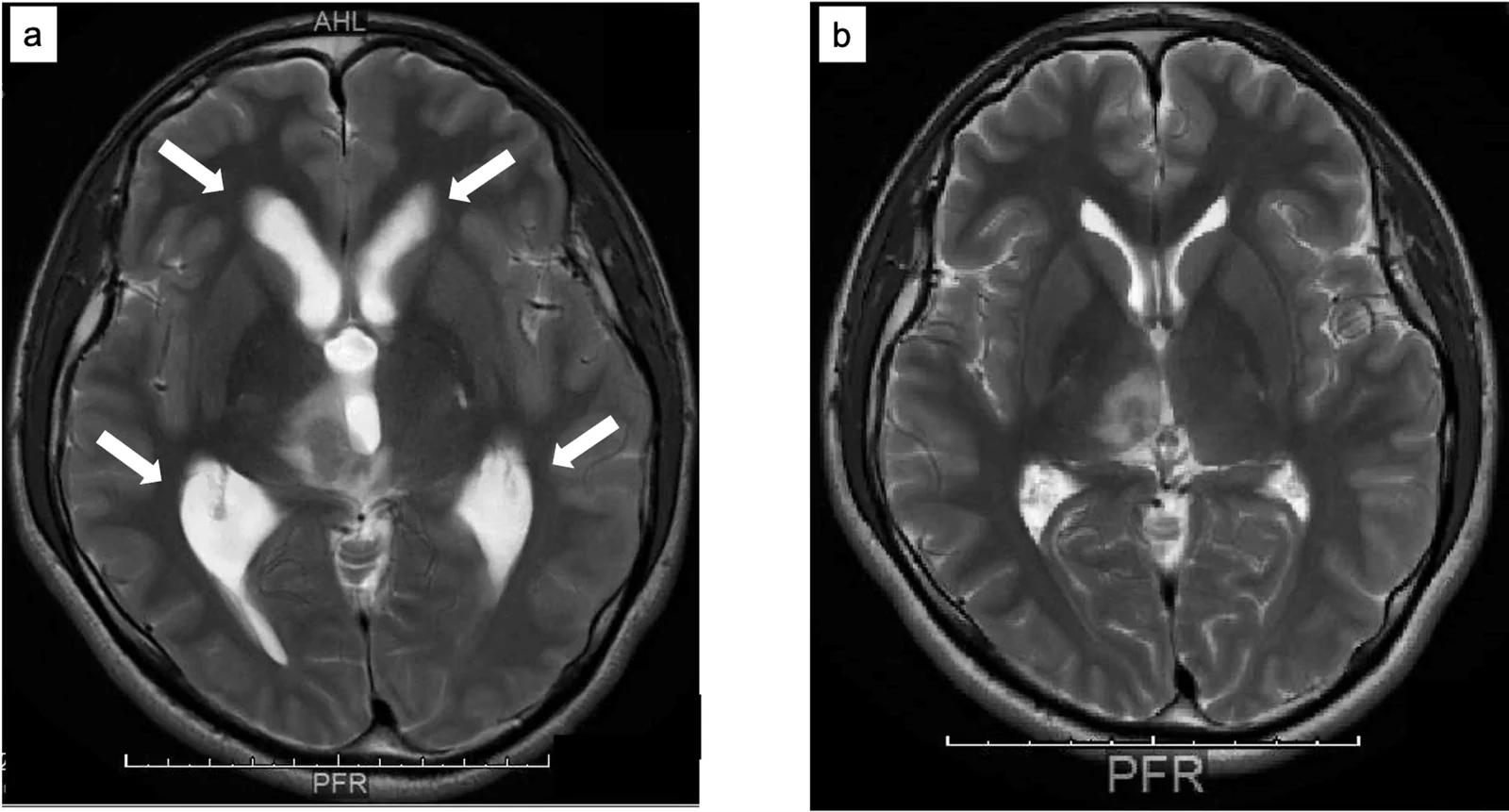
Brain magnetic resonance imaging (MRI) (T2-weighted images) (a) At the initial neurosurgical consultation; (b) one month after neurosurgery. At the initial consultation, narrowing of the cerebral aqueduct and hydrocephalus (white arrows) that were caused by a lobulated mass extending from the right midbrain to the right thalamus were observed (a). One month after surgery, the hydrocephalus had resolved (b).

The patient received carboplatin and etoposide (CARE) treatment in the second, third, and fourth months post-surgery, whole-ventricle radiotherapy (24 Gy/15 fractions) in the fifth and sixth months, and ifosfamide (IFO), cisplatin (CDDP), and etoposide (VP-16) (ICE) treatment in the seventh, ninth, and 11th months. No recurrence of the brain tumor has been observed since then. By the one-year mark post-surgery, the neuroretinitis had resolved (Figure 7), and OCT showed no abnormal findings (Figure 8).

**Figure 7:**
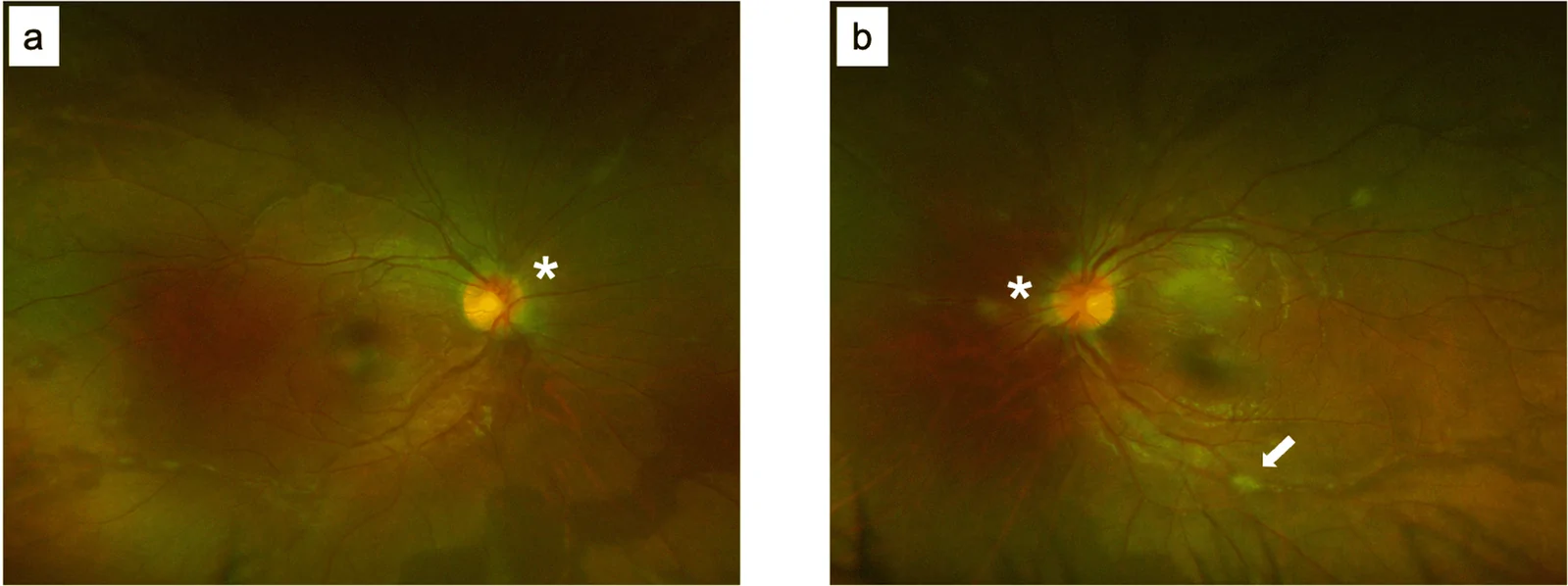
Color fundus photographs one year after neurosurgery (a) Right eye; (b) left eye. Although the optic disc margins remained slightly blurred in both eyes, the redness and swelling had improved (white asterisks). Exudates along the inferotemporal vascular arcade in the left eye had decreased in size.

**Figure 8:**
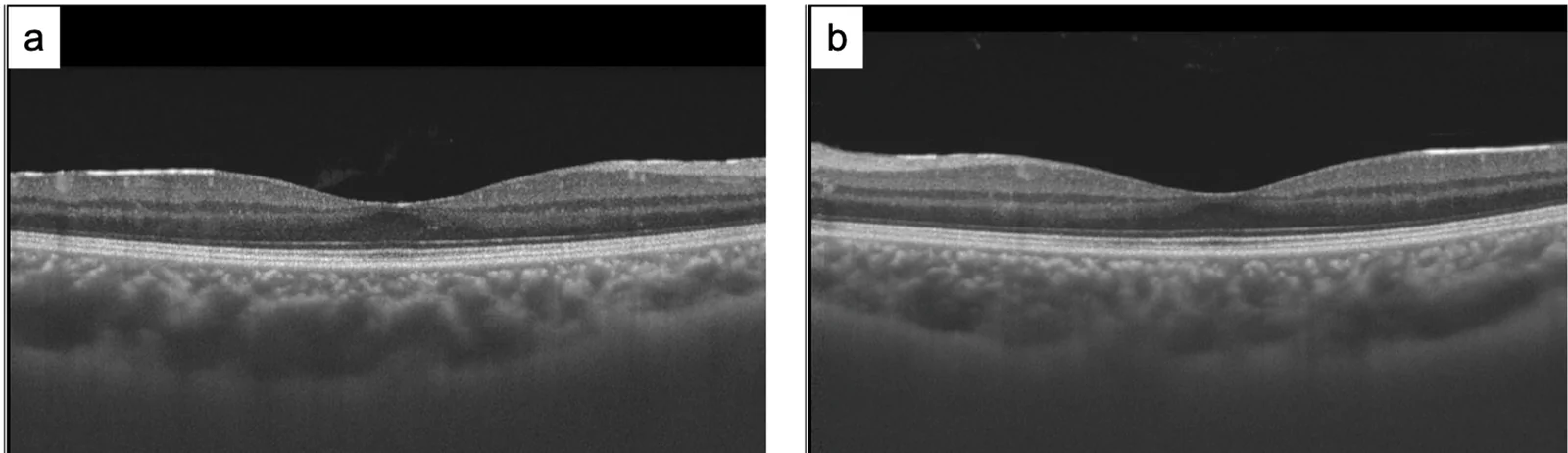
Optical coherence tomography (OCT) images one year after neurosurgery (a) Right eye; (b) left eye. No serous retinal detachment or intraretinal fluid was observed in either eye.

At the final follow-up visit five years post-surgery, uncorrected visual acuity was 1.5 in both eyes; no worsening of neuroretinitis findings was observed during the follow-up period (Figure 9), and OCT findings remained normal (Figure 10).

**Figure 9:**
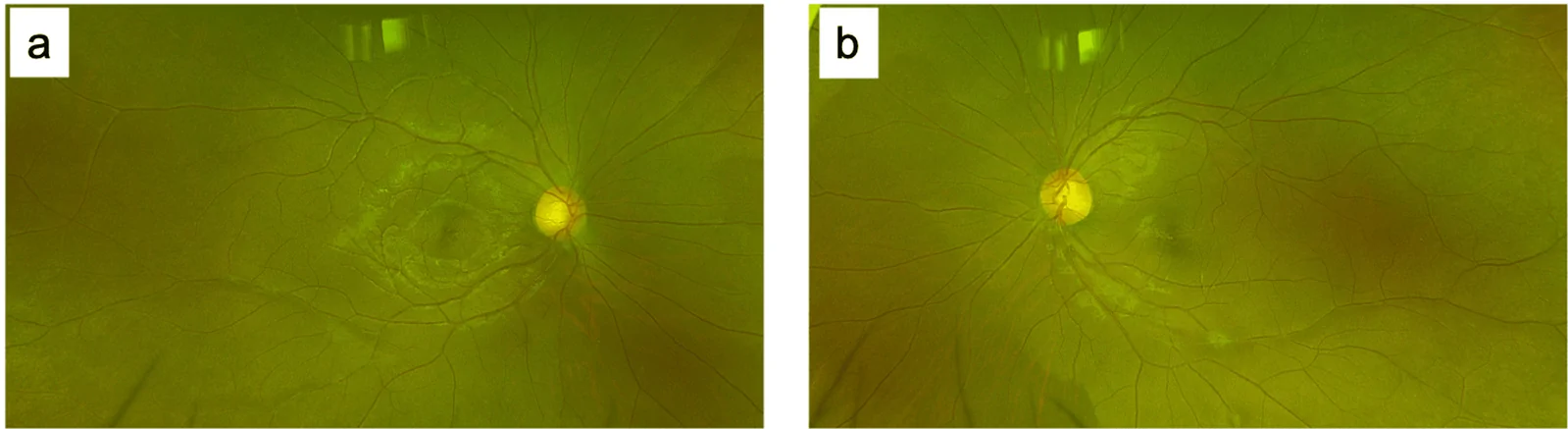
Color fundus photographs five years after the initial consultation (a) Right eye; (b) left eye. No obvious abnormalities of the optic disc were observed in either eye. Exudates along the inferotemporal vascular arcade in the left eye had further decreased in size.

**Figure 10:**
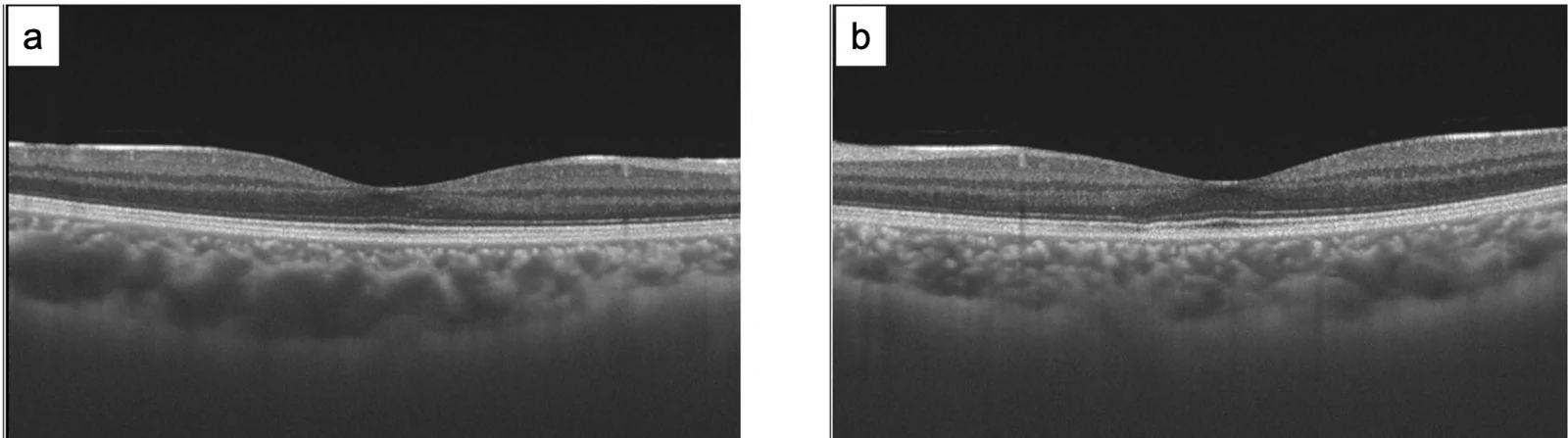
Optical coherence tomography (OCT) images five years after the initial consultation (a) Right eye; (b) left eye. No serous retinal detachment or intraretinal fluid was observed in either eye.

## Discussion

In the present case, as with previously reported cases of masquerade syndrome caused by germinoma, the patient presented with neuroretinitis. When neuroretinitis is observed, infectious causes such as tuberculosis [11], toxoplasmosis [12], toxocariasis [13], and bartonellosis [14] are suspected. In the absence of systemic immune abnormalities, the condition is often unilateral [15]. However, bilateral neuroretinitis has been reported in patients with compromised systemic immune status, such as those undergoing treatment with biological agents [16]. Our patient was a healthy young individual who tested negative for HIV antibodies, and blood test results showed no findings suggestive of immune abnormalities.

Although the patient presented with refractory neuroretinitis, no significant abnormalities were detected in either eye by Goldmann perimeter, and-in retrospect-intracranial evaluation using computed tomography (CT) or MRI was not performed. While there was no marked worsening of the neuroretinitis during the nine months following the initial visit, the persistence of inflammation should have raised suspicion of a masquerade syndrome. Fortunately, the onset of headache in this case led to the discovery of a germinoma. Previous reports indicate that cases presenting initially with retinal findings often require several months or longer before a germinoma is diagnosed [9,10]. This delay in diagnosis is likely attributable to the fact that germinomas are relatively rare as the underlying cause of masquerade syndromes. When neuroretinitis is observed in a young patient and infectious causes have been ruled out-particularly if the condition is bilateral and characterized by fluctuating symptoms-it is crucial to include intracranial germinoma in the differential diagnosis.

Neuroretinitis is not a common manifestation of masquerade syndrome [1]. However, it is frequently observed in cases of masquerade syndrome associated with germinoma [6-10]. Elevated intracranial pressure is considered to play a major role in the development of this neuroretinitis. Previous reports have noted that optic disc swelling-which failed to resolve with systemic steroid administration or the resection of a thymic germinoma-improved following treatment for an intracranial germinoma [10]. Indeed, in our case, performing an ETV resulted not only in the resolution of hydrocephalus one month post-surgery but also in the improvement of neuroretinitis. Thus, elevated intracranial pressure appears to play a significant role in the pathogenesis of masquerade syndrome associated with intracranial germinoma. Together with the fact that a pronounced peritumoral lymphocytic and granulomatous immune reaction [17] can occasionally mimic intraocular inflammation, neuroretinitis is considered to stem from inflammation related to leptomeningeal infiltration of tumor and elevated intracranial pressure.

## Conclusions

When ocular inflammatory diseases-such as uveitis-are observed, one should consider the possibility of masquerade syndromes associated with either neoplastic or non-neoplastic conditions. Masquerade syndromes presenting with neuroretinitis are rare, making them difficult to include in the differential diagnosis. However, in young patients presenting with neuroretinitis in the absence of infection or immunodeficiency, the possibility of intracranial tumors, such as germinoma, should be kept in mind during clinical management. Thus, persistent, bilateral, or fluctuating neuroretinitis with negative infectious investigations may warrant neuroimaging and consideration of an intracranial masquerade process.
